# Suffruticosol B Is an Osteogenic Inducer through Osteoblast Differentiation, Autophagy, Adhesion, and Migration

**DOI:** 10.3390/ijms232113559

**Published:** 2022-11-04

**Authors:** Hyung-Mun Yun, Joon Yeop Lee, Bomi Kim, Kyung-Ran Park

**Affiliations:** 1 Department of Oral and Maxillofacial Pathology, School of Dentistry, Kyung Hee University, Seoul 02447, Korea; 2 National Development Institute for Korean Medicine, Gyeongsan 38540, Korea; 3 Gwangju Center, Korea Basic Science Institute (KBSI), Gwangju 61751, Korea

**Keywords:** osteoblasts, phytomedicine, *Paeonia suffruticosa*, RUNX2, Suf-B

## Abstract

Suffruticosol B (Suf-B) is a stilbene found in *Paeonia suffruticosa* ANDR., which has been traditionally used in medicine. Stilbenes and their derivatives possess various pharmacological effects, such as anticancer, anti-inflammatory, and anti-osteoporotic activities. This study aimed to explore the bone-forming activities and mechanisms of Suf-B in pre-osteoblasts. Herein, >99.9% pure Suf-B was isolated from *P. suffruticosa* methanolic extracts. High concentrations of Suf-B were cytotoxic, whereas low concentrations did not affect cytotoxicity in pre-osteoblasts. Under zero levels of cytotoxicity, Suf-B exhibited bone-forming abilities by enhancing alkaline phosphatase enzyme activities, bone matrix calcification, and expression levels with non-collagenous proteins. Suf-B induces intracellular signal transduction, leading to nuclear RUNX2 expression. Suf-B-stimulated differentiation showed increases in autophagy proteins and autophagosomes, as well as enhancement of osteoblast adhesion and transmigration on the ECM. These results indicate that Suf-B has osteogenic qualities related to differentiation, autophagy, adhesion, and migration. This also suggests that Suf-B could have a therapeutic effect as a phytomedicine in skeletal disorders.

## 1. Introduction

The tree peony *Paeonia suffruticosa* ANDR. has been cultivated for medicinal uses for nearly 2000 years [1]. Extracts of the species contain bioactive compounds such as stilbene monoterpene, and paeonol compounds [2,3,4,5,6]. Stilbenes exhibit pharmacological properties against various ailments, such as infection, inflammation, cancer, and bone diseases [7,8,9,10,11]. Suffruticosol B (Suf-B), extracted from *P. suffruticosa*, was originally reported for its pharmacological effects as an ecdysteroid antagonist [12]. It has also been reported that Suf-B is cytotoxic and exhibits antitumor properties in the human liver, colon cancer, and promyelocytic leukemia [13,14,15]. In addition, the pharmacological effects of Suf-B suggest that it is a natural source of antioxidants and hypoglycemics [16,17]. However, the beneficial effects of Suf-B on osteoblast differentiation and osteogenic activity have not yet been elucidated.

Bone tissue is a mineralized tissue that is modulated by complex events during its lifetime [18,19]. For instance, mineralized tissue cells such as osteoblasts differentiate from mesenchymal stem cells and induce the development, formation, and repair of the mineralized tissue [20]. Skeletal diseases, such as periodontal disease and osteoporosis, occur due to the dysregulation of such physiological functions in the bone-formation process that maintain skeletal integrity [21,22,23]. Research on osteoblasts, from biological mechanisms to therapeutic strategies, has improved our understanding of the mechanisms that regulate the differentiation of osteoblasts and maintenance of bone structure and strength [22,24,25]. However, only a few safe and effective medications stimulate osteoblast differentiation and biological activity. Thus, it is important to explore the bioactivity of natural compounds in osteoblast differentiation and behavior.

In the present study, we purified >99.9% Suf-B from the fruits of *P. suffruticosa* and investigated its effects on cell viability in the MC3T3-E1 pre-osteoblasts. We further attempted to determine the biological activities and mechanisms of Suf-B-mediated differentiation, calcification, autophagy, adhesion, and migration.

## 2. Results

### 2.1. Isolation of Suf-B from P. suffruticosa Fruits

**Figure 1:**
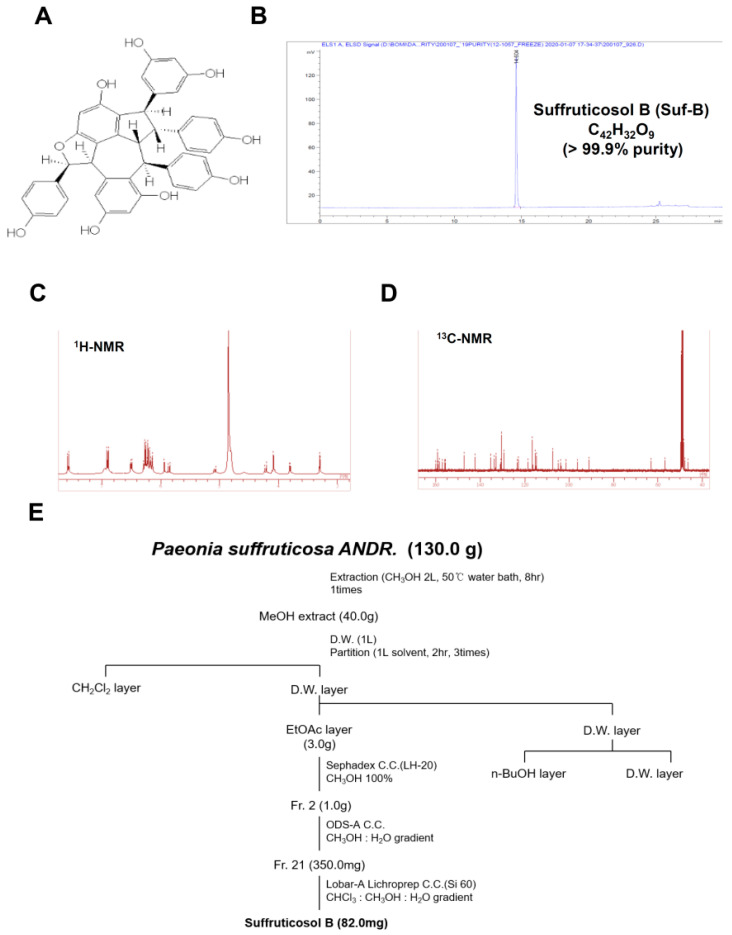
Isolation of Suffruticosol B (Suf-B): (**A**) Suf-B structure; (**B**) HPLC chromatogram, C_42_H_32_O_9_, >99.99% purity; (**C**,**D**) ^1^H NMR (**C**) and ^3^C NMR (**D**); and (**E**) isolation roadmap of Suf-B.

The chemical structure and high-performance liquid chromatography (HPLC) of Suf-B (>99.99%, brown powder, C_42_H_32_O_9_) isolated from dried fruits of *P. suffruticosa* are shown in Figure 1A,B. Figure 1C,D show the nuclear magnetic resonance (NMR) of Suf-B. The process of isolating Suf-B from *P. suffruticosa* is depicted in Figure 1E.

### 2.2. Suf-B Facilitates Osteoblast Differentiation and Mineralization

**Figure 2:**
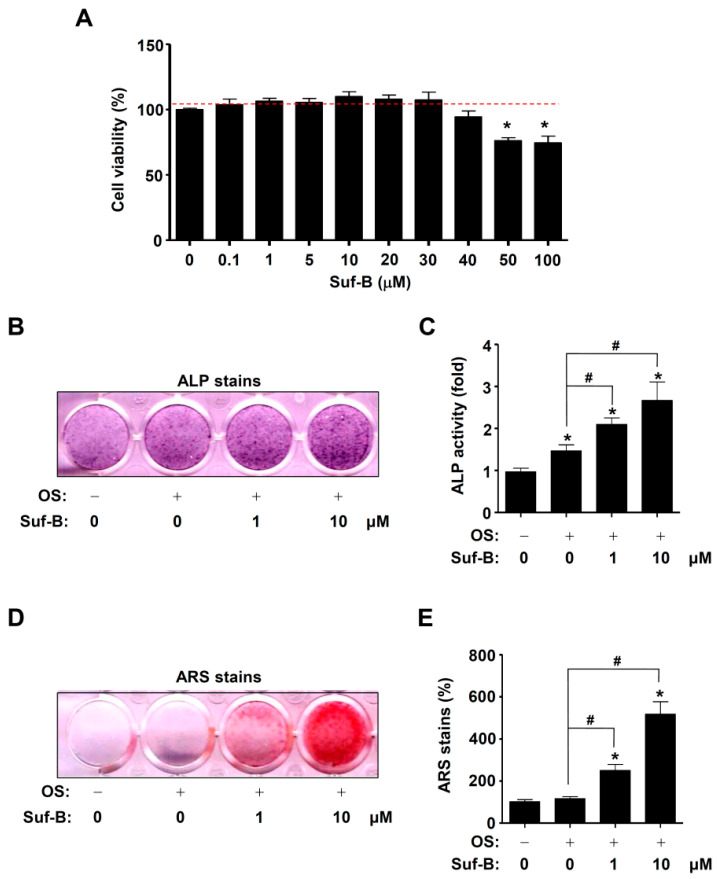
Effects of Suf-B on cytotoxicity, ALP, and calcification. (**A**) 1–100 μM Suf-B was treated for 24 h in pre-osteoblasts, and cytotoxicity was measured by using a cell viability assay. (**B**,**C**) Early differentiation was detected by ALP stains (**B**) and ALP activity (**C**). (**D**,**E**) Matrix calcification was stained by ARS (**D**), and the levels were detected by using a spectrophotometer (**E**). Note: * *p* < 0.05 compared with the control; # *p* < 0.05 compared with OS. Data are representative results of three experiments.

Pre-osteoblasts were treated with 0.1–100 μM Suf-B to investigate its effects on cell viability. Suf-B showed no cytotoxic effects at 0.1–40 μM, whereas it showed cytotoxic effects at 50–100 μM (Figure 2A). The biological activity of non-cytotoxic concentrations (1–10 μM) of Suf-B was further investigated. Osteoblast differentiation was induced for 7 days, and alkaline phosphatase (ALP) staining was conducted to monitor early differentiation. Staining images captured by the scanner showed that Suf-B promoted differentiation compared to an osteogenic supplement medium OS (Figure 2B). Enzymatic activity was measured by using an ELIZA reader, and the results statistically validated Suf-B-stimulated early osteoblast differentiation (Figure 2C), whereas Suf-B alone did not affect the osteoblast differentiation (Supplementary Figure S1). Late osteoblast differentiation was analyzed by using an alizarin red S staining (ARS) assay 21 days after inducing osteoblast differentiation to monitor bone-matrix mineralization. The ARS results showed that Suf-B promoted mineralization by late osteoblast differentiation compared with OS (Figure 2D). This was further validated by quantifying the ARS staining (Figure 2E).

### 2.3. Suf-B Facilitates BMP2-Smad1/5/8 Signaling and MAPKs Molecules 

**Figure 3:**
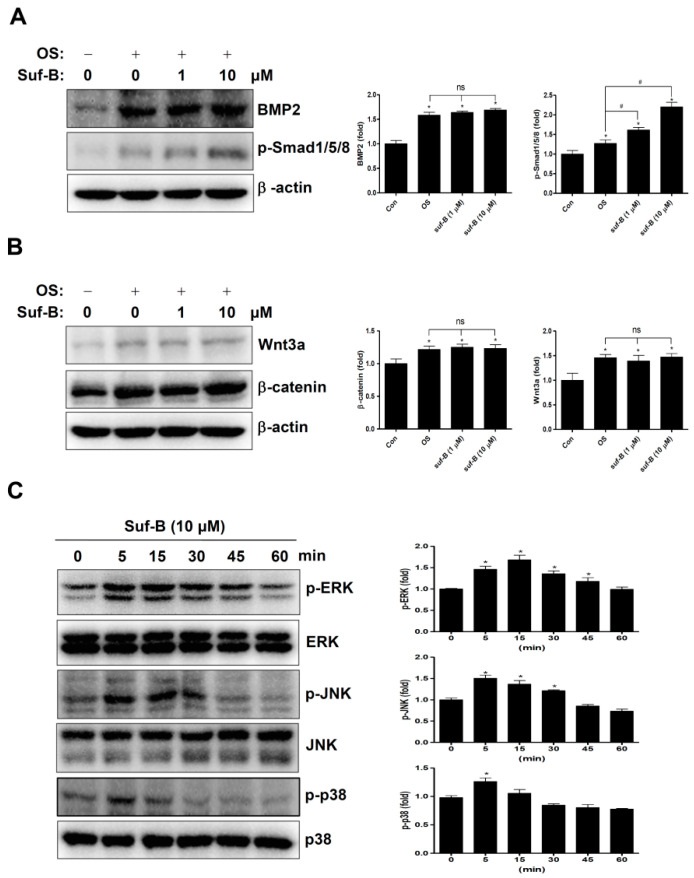
Effects of Suf-B on the osteogenic pathways. (**A**,**B**) The osteogenic pathways were determined by Western blot analysis with antibodies against BMP2, p-Smad1/5/8 (**A**), Wnt3a, and β-catenin (**B**). β-actin levels were detected as a loading control in total proteins. (**C**) p-ERK, ERK, p-JNK, JNK, p-p38, and p38 proteins were determined by Western blot analysis. Note: * *p* < 0.05 compared with the control. # *p* < 0.05 compared with OS. Data are representative results of three experiments.

To investigate the biological mechanisms underlying the osteogenic activity of Suf-B, we explored the main signaling molecules associated with osteoblast differentiation. We found that Suf-B enhanced the levels of phospho-Smad1/5/8, which is a core BMP2 signaling molecule, (Figure 3A), but did not affect the levels of Wnt3a and β-catenin compared with OS (Figure 3B). Suf-B also enhanced the expression of mitogen-activated protein kinases (MAPKs), including ERK, JNK, and p38 (Figure 3C), suggesting that Suf-B promotes BMP2, Smad1/5/8, and MAPKs during osteoblast differentiation.

### 2.4. Suf-B Facilitates RUNX2 Expression and Autophagy in Osteoblast Differentiation

**Figure 4:**
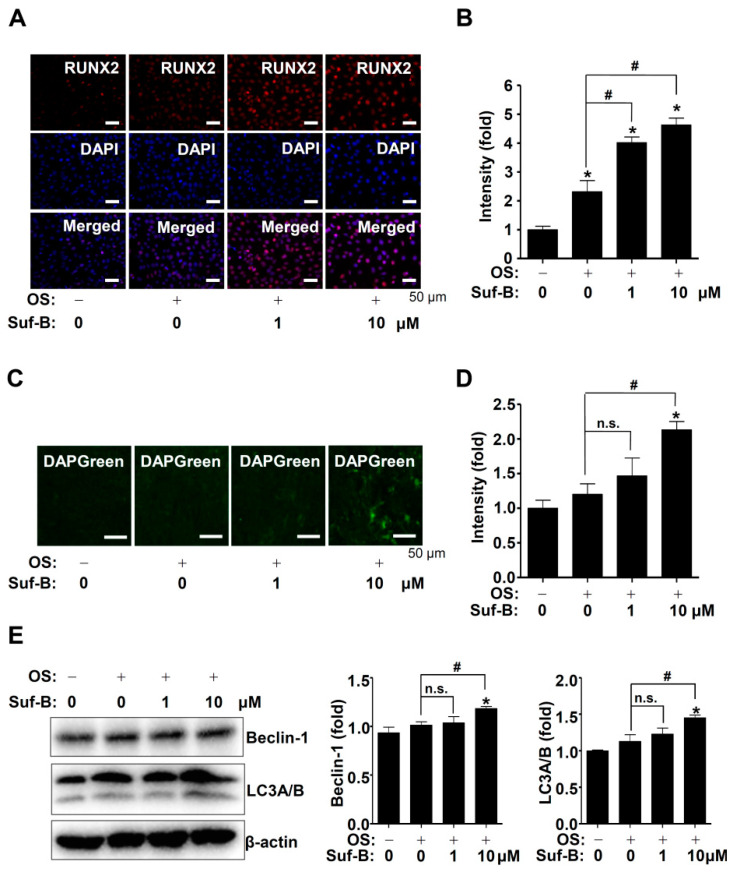
Effects of Suf-B on RUNX2 and autophagy. (**A**,**B**) The nuclear RUNX2 levels (red) were analyzed by using a fluorescence microscope. DAPI (blue) is a nuclear marker. The intensity was expressed on a graph (**B**). (**C**,**D**) Autophagosome formation was detected by using a fluorescence microscope (**C**), and the intensity was expressed on a graph (**D**). (**E**) Western blotting of Beclin-1, LC3A/B, and β-actin levels. Note: * *p* < 0.05 compared with the control; # *p* < 0.05 compared with OS. Data are representative results of three experiments.

BMP2-Smad1/5/8 and MAPKs are upstream signaling molecules that are involved in key transcription factors, such as runt-related transcription factor 2 (RUNX2), during the osteogenic processes. Thus, we further explored the effect of Suf-B on nuclear RUNX2 levels, using immunocytochemistry, and the results showed that Suf-B increased the expression levels of RUNX2 in the nucleus compared to OS (Figure 4A,B). Furthermore, we investigated autophagosome formation because RUNX2 is involved in autophagy during osteoblast differentiation. Autophagosome formation was detected by using DAPGreen, which showed that 10 μM Suf-B induced increased formation of autophagic vacuoles (Figure 4C,D). Moreover, Suf-B slightly increased the levels of Beclin-1 and LC3A/B (Figure 4E), indicating that the osteogenic effects of Suf-B are mediated by RUNX2 and autophagy. 

### 2.5. Suf-B Facilitates Osteoblast-Mediated Bone-Forming Phenotypes

**Figure 5:**
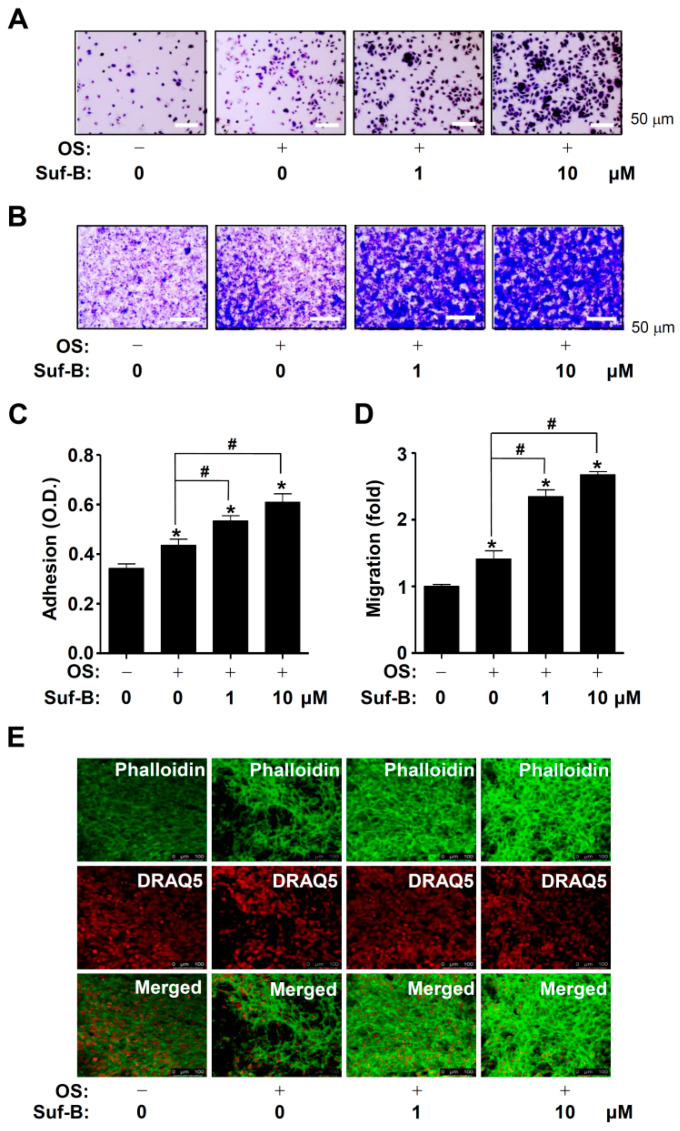
Effects of Suf-B on adhesion and migration. Cell adhesion on ECM was detected by using a light microscope (**A**); the level was measured with an ELIZA reader and expressed on a graph (**C**). Cell migration across ECM was detected by using a light microscope (**B**). The stained level was measured with an ELIZA reader and expressed on a graph (**D**). (**E**) F-actin polymerization (phalloidin, green) was detected by using an intravital multi-photon microscope system (IMPM). DRAQ5 (red) is a nuclear marker. Note: * *p* < 0.05 compared with the control; # *p* < 0.05 compared with OS. Data are representative results of three experiments.

Finally, we examined whether Suf-B influences adhesion and transmigration during osteoblast differentiation. Matrigel-coated plates revealed that Suf-B significantly promoted cell adhesion to the extracellular matrix (ECM) compared to OS (Figure 5A,C). Subsequently, a transmigration assay also showed that Suf-B significantly facilitated transmigration across the Matrigel-coated membrane (Figure 5B,D). In addition, we validated F-actin polymerization during osteoblast differentiation (Figure 5E).

## 3. Discussion

Osteoblasts mineralize unmineralized osteoid and matrix through processes such as differentiation, adhesion, and migration thus leading to skeletal formation, development, and repair [26,27]. Osteoblasts are also involved in bone diseases, which cause fragile bones with poor density and strength leading to fractures [23,28]. In the present study, we demonstrated the bioactivity of Suf-B in osteoblast differentiation and matrix mineralization via the osteogenic signaling pathway and RUNX2 expression.

As a transcription factor, RUNX2 increases the expression of ALP during osteoblast differentiation [29,30]. ALP serves as a well-known marker for early osteoblast differentiation [31,32,33] and is also known as the key initial enzyme for osteogenesis during bone formation [31,32,33]. Its osteogenic activity hydrolyzes inorganic pyrophosphate and organic phosphomonoesters and causes the synthesis of hydroxyapatite and deposition of minerals, leading to bone formation [34,35]. ALP deletion results in abnormal bone formation and spontaneous fracture [36]. In the present study, we found that Suf-B promoted ALP activity during osteoblast differentiation and subsequently promoted mineralization during osteoblast maturation. In addition, we investigated the osteogenic effects of Suf-B alone and found that Suf-B alone fails to stimulate the osteoblast differentiation. Therefore, our findings suggest that Suf-B enhances osteogenic activities during osteoblast differentiation under osteogenic condition, but not Suf-B alone.

Various natural compounds have been reported as novel osteogenic inducers that promote intracellular signal transduction [37,38,39,40,41,42,43]. BMP2 signaling is a key target in osteoblast differentiation, osteoid formation, and calcification. Genetic mutations in BMP2 are involved in osteoporotic risk, including low bone mineral density and high fractures both before and after menopause [44]. Osteogenic compounds activate BMP2 signaling proteins, which promote the RUNX2 transcription factor [41,42,45,46]. In this study, we demonstrated that Suf-B enhances BMP2-Smad1/5/8-RUNX2 signal transduction in osteoblast differentiation. Several studies have shown that Wnt signaling induces BMP2 expression, which is closely associated with osteoporotic risk [41]. Kirenol has been reported to promote osteoblast differentiation via BMP and Wnt in MC3T3-E1 preosteoblasts, suggesting that it may be a candidate target for patients with osteoporosis [47]. We recently demonstrated that anthraquinone from *Rubia cordifolia* enhances osteogenic processes via the BMP and Wnt pathways [48]. In addition, MAPKs are associated with BMP2 and RUNX2 expression and osteoblast differentiation [41,49]. BMP2 also activates MAPKs signaling via a non-canonical pathway for osteoblast differentiation [50]. In the present study, we further investigated Wnt and MAPKs signaling proteins and demonstrated that Suf-B is not involved in Wnt signaling, but it does activates MAPKs signaling. Therefore, our evidence suggests that Suf-B exerts osteogenic effects through BMP2-induced signaling and RUNX2 expression, leading to osteogenic gene expression.

Recent studies suggest that autophagy promotes osteoblast differentiation, survival, and function, as well as bone diseases [51,52,53]. The flavonoid kaempferol has been reported to have various osteogenic activities and the ability to enhance autophagy to promote differentiation in MC3T3-E1 pre-osteoblasts [54]. Stilbenes and derivative compounds have also been reported to regulate autophagy [55]. Thus, we explored whether Suf-B affects autophagy during osteoblast differentiation and demonstrated that high-dose Suf-B promotes autophagosome formation. In addition, we investigated osteoblast adhesion and migration phenotypes because they are required for osteoblast-mediated bone formation [56,57]. In this study, we found that Suf-B potentiated osteoblast adhesion and migration in the ECM. Osteoblasts migrate and attach to form osteoids and mineralized tissues [58,59,60]. To further validate the Suf-B-mediated phenotypes, we monitored cytoskeletal changes and demonstrated that Suf-B increases F-actin polymerization during osteoblast differentiation. The inhibition of F-actin polymerization has also been reported to suppress osteoblast differentiation [61,62]. Therefore, our evidence suggests that Suf-B modulates autophagy, adhesion, migration, cytoskeletal changes, and subsequent differentiation and maturation.

## 4. Materials and Methods

### 4.1. Material, Procedures, and Isolation

The P838 voucher specimen (*P. suffruticosa* dried fruit) was deposited at the Natural Products Bank (NIKOM, Gyeongsan, Korea). The isolation of Suffruticosol B (Suf-B) was carried out in the same experimental procedure as previously described [63], and 82 mg Suf-B (99.99%, brown powder, C_42_H_32_O_9_) was isolated from 130.0 g *P. suffruticosa* dried fruit.

### 4.2. Pre-Osteoblast and Differentiation

MC3T3-E1 pre-osteoblasts were obtained from the American Type Culture Collection (Manassas, VA, USA) and cultured in α-minimum essential medium without L-ascorbic acid (L-AA) (WELGEME, Inc., Gyeonggido, Korea), containing 10% fetal bovine serum and 1× antibiotic–antimycotic (Thermo Fisher Scientific, Waltham, MA, USA), in a CO_2_ incubator MCO-18AC (Panasonic, Osaka, Japan). Osteoblast differentiation was initiated by using an osteogenic supplement medium OS containing 10 mM β-glycerophosphate (Sigma-Aldrich, St. Louis, MO, USA) and 50 μg/mL L-AA (Sigma-Aldrich) in a CO_2_ incubator. The OS was changed every 2 days for differentiation. 

### 4.3. MTT Assay

The MTT assay was performed as previously described [63]. Briefly, MTT (Sigma-Aldrich) solution (5 mg/mL stock in 1× PBS) was directly added to MC3T3-E1 pre-osteoblasts for 2 h. Formazan was dissolved in 200 μL of 100% dimethyl sulfoxide (DMSO) (Sigma-Aldrich), and the absorbance was detected at a 540 nm wavelength in a Multiskan GO Microplate Spectrophotometer (Thermo Fisher Scientific).

### 4.4. ALP and ARS Assays

ALP staining and activity were analyzed by using an ALP reaction solution (Takara Bio Inc., Tokyo, Japan) and ALP activity colorimetric assay kit (Biovision, Milpitas, CA, USA), respectively, as previously described [63]. ARS was analyzed by using a 2% ARS (Sigma-Aldrich) solution (pH 4.2) to monitor mineralization, as previously described [49,63].

### 4.5. Western Blot Analysis

Western blot analysis was performed as previously described [64,65]. Briefly, protein concentration was determined by using Bradford reagent (Bio-Rad, Hercules, CA, USA), and the resolved gel was transferred to a PVDF membrane (Millipore, Bedford, MA, USA). After incubation with primary and secondary antibodies, the PVDF membrane was treated with an ECL solution (Millipore, Bedford, MA), and immunoreactive bands were detected by using a ProteinSimple machine (ProteinSimple Inc., Santa Clara, CA, USA).

### 4.6. Immunocytochemistry

Immunocytochemistry was carried out as previously described [63].

### 4.7. Autophagy Detection

An autophagy detection kit (Dojindo, Kumamoto, Japan) was used to monitor autophagosome formation, which was observed by using a fluorescence microscope. Autophagic signaling proteins were detected by Western blot analysis, as described previously [63,66].

### 4.8. Adhesion and Migration Assays

Matrigel solution (Corning Life Sciences, Tewksbury, MA, USA) and 0.5% crystal violet (Sigma-Aldrich) were used, as previously described [49,67]. The adhesion and migration of cells were detected by using a Multiskan GO Microplate Spectrophotometer (Thermo Fisher Scientific) and a light microscope.

### 4.9. Phalloidin and DRAQ5 Staining

Phalloidin (Thermo Fisher Scientific) was used to monitor F-actin polymerization, and DRAQ5 (Thermo Fisher Scientific) was used to detect the nucleus, which was observed by using an intravital multi-photon microscope system (IMPM) at Gwangju Center, Korea Basic Science Institute (KBSI).

### 4.10. Statistical Analysis

Prism Version 5 (GraphPad Software, Inc., San Diego, CA, USA) was used for statistical analysis. Statistical significance was assessed by using one-way ANOVA with post hoc analysis (*p* < 0.05), and data are reported as mean ± SEM.

## 5. Conclusions

We provide convincing evidence that Suf-B is a novel osteogenic inducer through BMP2-mediated signaling pathways in pre-osteoblasts. Plant-derived natural compounds have therapeutic effects against various diseases, with fewer side effects than chemical compounds [19]. Although in vivo animal studies are required to explore Suf-B-mediated bone formation, our in vitro results suggest that Suf-B is a potential candidate for osteoblast-mediated anabolic drugs based on biological mechanisms through the intracellular signaling transduction pathway.

## Data Availability

The data that support the findings of this study are available from thecorresponding author upon reasonable request.

## Supplementary Materials

The following supporting information can be downloaded at: https://www.mdpi.com/article/10.3390/ijms232113559/s1.

## Abbreviations

ALP	Alkaline phosphatase
ARS	Alizarin red S staining
L-AA	L-ascorbic acid
MAPKs	Osteogenic supplement medium
*P. suffruticosa*	*Paeonia suffruticosa*
RUNX2	Runt-related transcription factor 2
Suf-B	Suffruticosol B
