# Supplementary material for: Suffruticosol B Is an Osteogenic Inducer through Osteoblast Differentiation, Autophagy, Adhesion, and Migration
Source: Int J Mol Sci. 2022 Nov 4;23(21):13559. doi: 10.3390/ijms232113559 (PMC9658763; doi:10.3390/ijms232113559)
Supplement: Supplementary file 1 [file ijms-23-13559-s001.zip › ijms-1962186-supplementary.pdf]

A

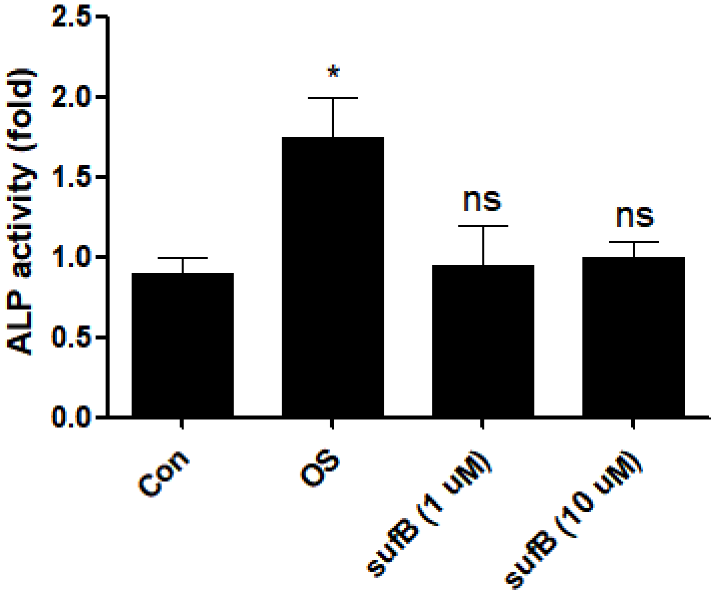

Supplementary Figure S1. Effects of Suf-B alone on osteoblast. (A) Suf-B was treated for 7 days in pre-osteoblasts, and osteoblast differentiation was detected by ALP activity. *, $p < 0.05$: compared with the control (Con).
